# The role of tumor-associated macrophages in glioma cohort: through both traditional RNA sequencing and single cell RNA sequencing

**DOI:** 10.3389/fonc.2023.1249448

**Published:** 2023-09-14

**Authors:** Yunan Hou, Wenjin Qiu, Yuanguo Ling, Xiaolan Qi, Jian Liu, Hua Yang, Liangzhao Chu

**Affiliations:** ^1^ Department of Neurosurgery, The Affiliated Hospital of Guizhou Medical University, Guiyang, Guizhou, China; ^2^ Key Laboratory of Endemic and Ethnic Diseases, Ministry of Education & Key Laboratory of Medical Molecular Biology of Guizhou Province, Guizhou Medical University, Guiyang, Guizhou, China; ^3^ Department of Neurosurgery, Guizhou Provincial People’s Hospital, Guiyang, Guizhou, China

**Keywords:** tumor-associated macrophages, glioma, PTX3, cell proliferation, prognostic indicator

## Abstract

Gliomas are the leading cause in more than 50% of malignant brain tumor cases. Prognoses, recurrences, and mortality are usually poor for gliomas that have malignant features. In gliomas, there are four grades, with grade IV gliomas known as glioblastomas (GBM). Currently, the primary methods employed for glioma treatment include surgical removal, followed by chemotherapy after the operation, and targeted therapy. However, the outcomes of these treatments are unsatisfactory. Gliomas have a high number of tumor-associated macrophages (TAM), which consist of brain microglia and macrophages, making them the predominant cell group in the tumor microenvironment (TME). The glioma cohort was analyzed using single-cell RNA sequencing to quantify the genes related to TAMs in this study. Furthermore, the ssGSEA analysis was utilized to assess the TAM-associated score in the glioma group. In the glioma cohort, we have successfully developed a prognostic model consisting of 12 genes, which is derived from the TAM-associated genes. The glioma cohort demonstrated the predictive significance of the TAM-based risk model through survival analysis and time-dependent ROC curve. Furthermore, the correlation analysis revealed the significance of the TAM-based risk model in the application of immunotherapy for individuals diagnosed with GBM. Ultimately, the additional examination unveiled the prognostic significance of PTX3 in the glioma group, establishing it as the utmost valuable prognostic indicator in patients with GBM. The PCR assay revealed the PTX3 is significantly up-regulated in GBM cohort. Additionally, the assessment of cell growth further confirms the involvement of PTX3 in the GBM group. The analysis of cell proliferation showed that the increased expression of PTX3 enhanced the ability of glioma cells to proliferate. The prognosis of glioblastomas and glioma is influenced by the proliferation of tumor-associated macrophages.

## Introduction

Gliomas are the primary cause of over 50% of all brain tumors, predominantly malignant. Gliomas with malignant characteristics usually have poor prognoses, recurrence rates, and mortality rates (1). The current treatment of gliomas relies mainly on surgical resection, postoperative adjuvant chemotherapy, targeted therapies, etc., but the therapeutic effect has not been satisfactory (2). The World Health Report categorizes gliomas into four grades, where grades II and III are referred to as diffuse low-grade gliomas (LGG), and grade IV gliomas are known as glioblastomas (GBM) (3). Studies have demonstrated that glioblastoma, a remarkably aggressive type of cancer, typically results in a median survival period of merely 16 months, in contrast to the lifespan of LGG patients which ranges from 1 to 15 years (4). Despite the fact that surgical removal, radiation therapy, and drug treatment are established therapies, glioma patients still exhibit resistance to existing medical interventions because of the extremely invasive characteristics of the disease (5). To develop successful therapeutic targets for glioma, it is crucial to examine the molecular mechanisms linked to the disease. As a result of recent advances in “omics”-based technologies, biomarker research has seen a surge of activity, especially in cancer research (6). Typically, a biomarker is defined as ‘a feature that is measured and assessed objectively to predict normal biological processes or pathogenic processes (7). Gliomas can be treated through three primary methods: surgical intervention, radiation therapy, and chemotherapy. The Neuro-Oncology Response Assessment Criteria are employed to evaluate the magnetic resonance imaging depiction of tumors in individuals with gliomas (8). Cancer is linked to various biomarkers, such as nucleic acids, proteins, sugars, lipids, small metabolites, cytogenetic factors, and cytokine factors (9).

The impact of aberrant glioma-associated signaling is also influenced by a intricate web of interactions between the tumor microenvironment (TME) and abnormal glioma-associated signaling (10). Besides endogenous signaling pathways, cancer cells also release substances that influence the role and makeup of the glioma tumor microenvironment (11). As a result, the cells within the tumor microenvironment (TME) have the ability to alter multiple biological aspects of the tumor, including growth, viability, movement, and evading the immune system (12). Gliomas have the highest number of tumor-associated macrophages (TAM), consisting of brain microglia and macrophages, within the TME (13). Macrophage activation is a defining feature of numerous diseases. Macrophages can be categorized into two different subtypes depending on the type of activation they undergo: classically stimulated M1 macrophages or alternatively stimulated M2 macrophages (14). M1 macrophages, which play a role in the immune system’s bactericidal and antitumor functions, release proinflammatory cytokines. In contrast, M2 macrophages release substances that reduce inflammation, remove waste, support the growth of new blood vessels, suppress the body’s defense system, promote the healing of wounds, and aid in the restoration of tissues (15).

Our objective in this study is to investigate the involvement of tumor-infiltrating macrophages (TIM) in a cohort of glioma cases. In the glioma cohort, the investigation of macrophage-associated genes was conducted using single-cell RNA sequencing. The multiple pathways enrichment analysis was involved for relative pathways. Also, the risk model was also involved for the exploration of the key genes in TAM.

## Methods

### Identification of TAM-related genes and glioma dataset from online databases

Several markers related to TAM have been identified in previous studies, such as C11orf45, CD68, CLEC5A, CYBB, FUCA1, GPNMB, HS3ST2, LGMN, MMP9, and TM4SF19. Furthermore, we acquired a dataset of RNA from individual cells through the Tumor Immune Single-cell Hub 2 (TISCH2) online database. Moreover, the transcriptomic information of individuals with glioma was acquired from the Cancer Genome Atlas (TCGA) repository.

### Single-sample gene set enrichment analysis in glioma cohort

We measured the extent of immune cell infiltration in individual samples by utilizing the ssGSEA function from the gsva package in R. The evaluation of immune cell infiltration in glioma tissues was conducted by utilizing the gene signature of immune cell populations in this analysis. Additionally, we utilized the CIBERSORT algorithm to compute the ratio of immune cells in glioma samples.To estimate the ratio of stromal and immune cells in the tumor samples, the ESTIMATE technique was utilized.

### Pathways enrichment analysis

In order to investigate various molecular mechanisms, we employed the R package called ‘clusterProfiler’ to conduct enrichment analyses of Gene Ontology (GO) and Kyoto Encyclopedia of Genes and Genomes (KEGG) using genes that were differentially expressed (DEGs).

### Differentially expressed gene analysis

Using the “limma” R package, we identified DEGs in glioma patients. Furthermore, the consensus cluster plus R package was utilized to perform DEG-based cluster analysis.

### Prognostic gene selection using Cox regression analysis and the least absolute shrinkage and selection operator method

Survival-associated key genes were identified through the implementation of univariate Cox regression analysis. To prevent overfitting and select essential genes, the LASSO Cox regression model was employed. LASSO regression was performed using the glmnet package in R. Feature selection was performed using multivariate Cox regression analysis, and genes with a p-value less than 0.05 were chosen.

### Pathway exploration using gene set enrichment analysis

The utilization of Gene Set Enrichment Analysis (GSEA) was implemented to detect possible pathways. Based on the median expression, the samples were categorized into high or low expression groups for key genes. Pathway analysis utilized datasets sourced from the Molecular Signature Database (MsigDB).

### Cell culture

Human glioma cell lines U251 and U87 were purchased from Beijing Zhongyuan Heju Biotechnology Co., Ltd. (Beijing, China). The cell lines were grown in Dulbecco’s Modified Eagle Medium (DMEM, Sigma, USA) with the addition of 10% fetal bovine serum (FBS, Gibco, USA) at a temperature of 37°C in the presence of 5% CO2.

### Cell Counting Kit-8 assay

The CCK-8 assay was used to assess cell viability. Cells were seeded into 96-well plates at a density of 5,000 cells per well and allowed to attach for 24 hours at 37°C with 5% CO2. After various treatments, 10 µl of CCK-8 solution was added to each well and the plates were incubated for an additional 2-4 hours at 37°C. The absorbance at 450 nm, which correlates with the number of viable cells, was then measured using a microplate reader.

### EdU staining

Glioma cells that underwent transfection were placed in each well of six-well tissue culture plates, with a density of 1 × 10^5^ cells per well. Following the indicated treatments, cell proliferation was tested using EdU Apollo-567 Detection Kit, and nuclear EdU and DAPI staining were observed under a fluorescence microscope.

### Statistical analysis

The R programming language was used for all statistical analyses. Kaplan-Meier survival curves were constructed using the Kaplan-Meier method. The R packages timeROC, survival, and survivalminer were utilized to perform a multivariate ROC analysis. For correlation analysis, a p-value below 0.05 was deemed to be statistically significant (16, 17).

## Results

### The glioma cohort was shown to have TAM through single-cell RNA sequencing

**Figure 1:**
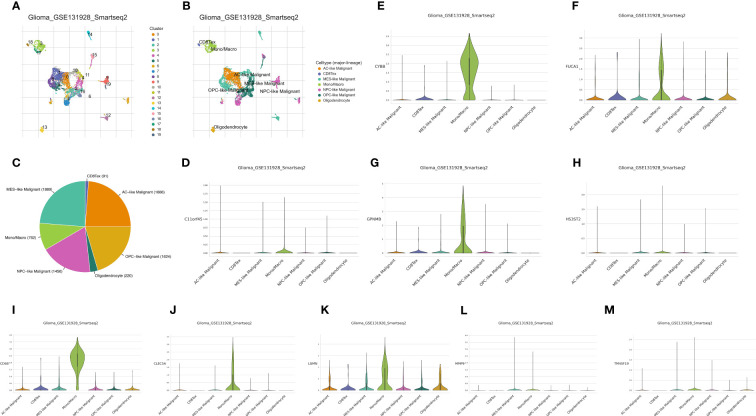
**(A)** The cell clustering analysis of single-cell RNA sequence; **(B)** The different cells involved in single-cell RNA sequence of glioma cohort; **(C)** The cell proportion of different cells in glioma cohort; The expression of C11orf45 **(D)**, CYBB **(E)**, FUCA1 **(F)**, GPNMB **(G)**, HS3ST2 **(H)**, CD68 **(I)**, CLEC5A **(J)**, LGMN **(K)**, MMP9 **(L)** and TM4SF19 **(M)** in glioma cohort.

**Figure 2:**
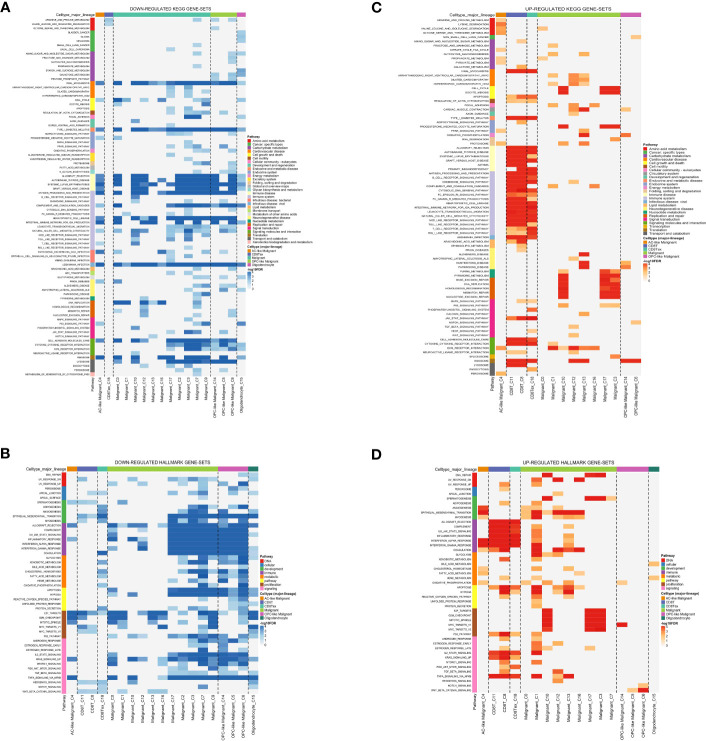
**(A)** The down-regulated KEGG gene set; **(B)** The down-regulated Hallmark gene set; **(C)** The up-regulated KEGG gene set; **(D)** The up-regulated Hallmark gene set.

Analysis of the glioma cohort using single-cell RNA sequencing identified various cell types, such as AC-like cancerous cells, CD8 T cells, MES-like cancerous cells, macrophages, NPC-like cancerous cells, OPC-like cancerous cells, and oligodendrocytes (**Figures 1A–C**). Macrophages showed notable enrichment of TAM-associated genes including C11orf45, CD68, CLEC5A, CYBB, FUCA1, GPNMB, HS3ST2, LGMN, MMP9, and TM4SF19 among others (**Figures 1D–M**). Significantly enriched KEGG terms identified through pathway enrichment analysis include the nod-like receptor signaling pathway, toll-like receptor signaling pathway, T cell receptor signaling pathway, and glutathione metabolism (**Figures 2A, C**). Furthermore, key routes such as the metabolism of fatty acids, the pathway of reactive oxygen species, and the early estrogen receptor were discovered to be significantly enriched (**Figures 2B, D**).

### The ssGSEA examination unveiled the scores associated with the immune system in the glioma group

**Figure 3:**
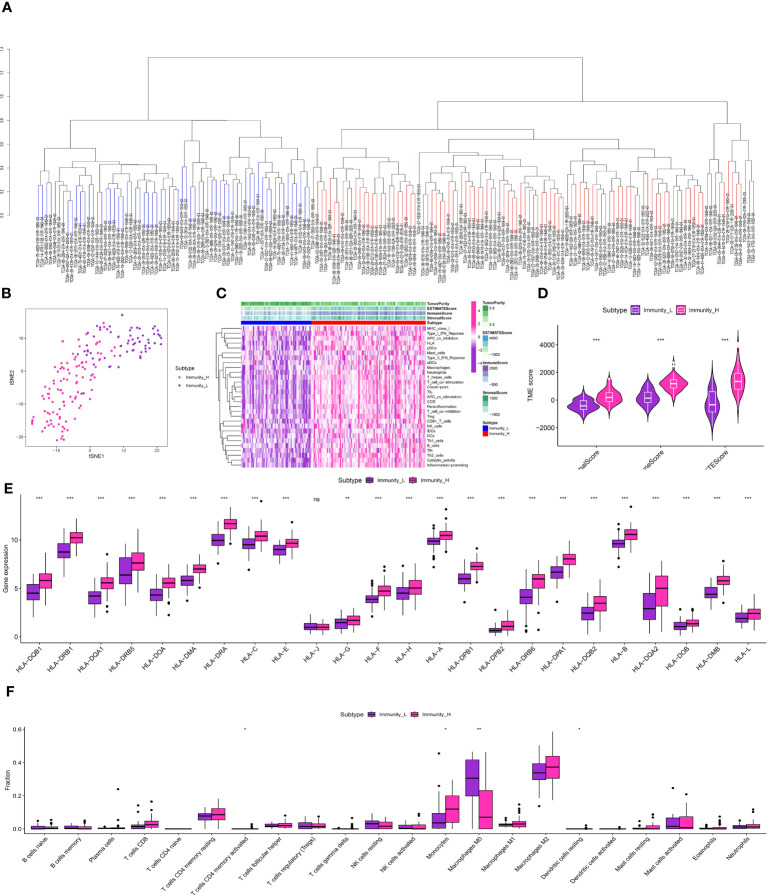
**(A)** The ssGSEA analysis in TCGA-GBM cohort; **(B)** The clustering analysis revealed the immune-related scores in TCGA-GBM cohort; **(C)** The heatmap demonstrated the immune-related scores and immune-related functions in TCGA-GBM cohort; **(D)** The correlation analysis between TME and immune-related scores; **(E)** The correlation analysis between HLA-related genes and immune-related scores; **(F)** The correlation analysis between immune-related cells and immune-related scores. * is equal to p < 0 .05. ** is equal to p < 0.01. *** is equal to p < 0.001. The ns is equal to no difference in data.

**Figure 4:**
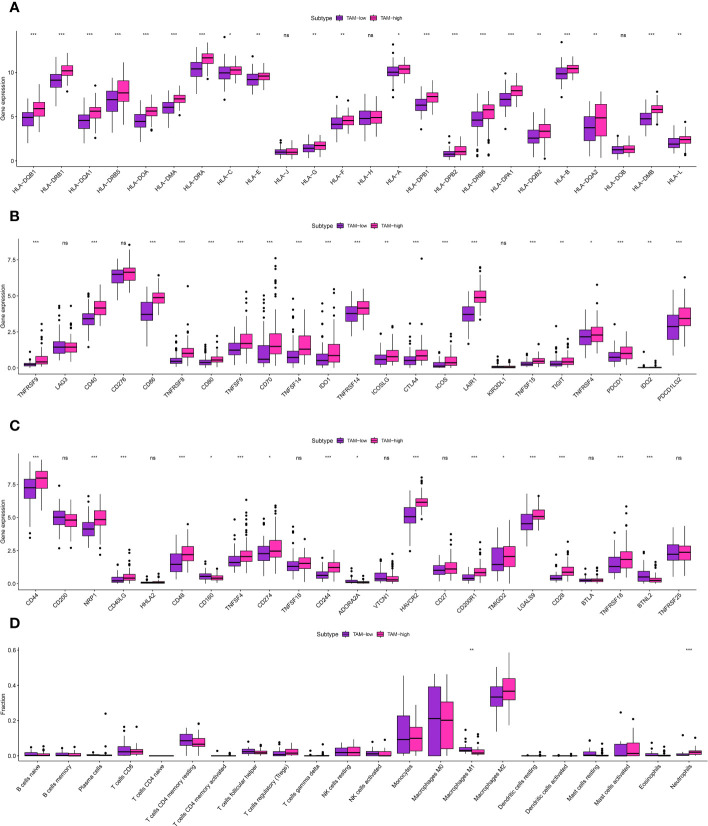
**(A)** The correlation analysis between TAM-related score and HLA-related genes; **(B, C)** The correlation analysis between TAM-related score and immune checkpoint-related genes; **(D)** The correlation analysis between TAM-related score and immune-related cells. * is equal to p < 0 .05. ** is equal to p < 0.01. *** is equal to p < 0.001. The ns is equal to no difference in data.

The glioma cohort underwent ssGSEA analysis to assess immune-related scores, TME-related scores, and immune-related functions in the GBM cohort, comprising 175 patients from TCGA.The GBM cohort was classified into IMMUNE-high and IMMUNE-low groups based on the scores related to the immune system and the scores related to the tumor microenvironment (TME) (**Figures 3A–D**).The analysis of correlation showed a positive relationship between the levels of expression of genes related to human leukocyte antigen (HLA) and scores related to the immune system (**Figure 3E**). In addition, analysis of immune cell infiltration revealed different patterns of distribution for activated CD4 T memory cells, monocytes, M0 macrophages, and dendritic cells in relation to the immune-related scores (**Figure 3F**). The clusters associated with TAM exhibited strong associations with cells related to the immune system, genes related to HLA, and genes related to immune checkpoints. The analysis of multiple correlations showed that increased immune-related scores were linked to higher levels of expression in genes related to HLA (**Figure 4A**). Genes associated with immune checkpoints showed notable reactions to immune-related scores, emphasizing their potential contribution to immune checkpoint treatment for glioma (**Figures 4B, C**). Moreover, immune cell infiltration analysis revealed differences in M1 macrophages and neutrophils between the TAM-high and TAM-low groups (**Figure 4D**).

### Identification of the TAM-related genes in glioma cohort

**Figure 5:**
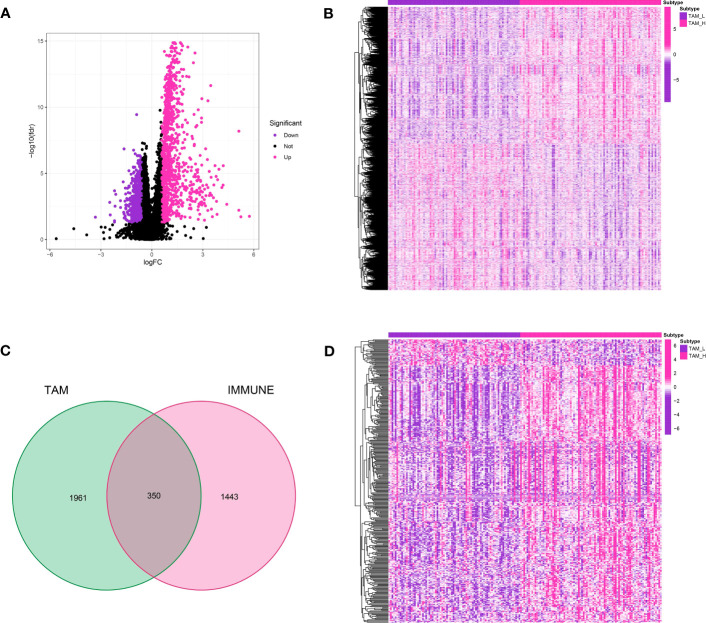
**(A)** The volcano map demonstrated the differentially expressed genes between TAM-high and TAM-low groups; **(B)** The heatmap demonstrated the expression level of key genes in glioma cohort; **(C)** The Venn diagram showed the immune-related genes in differentially expressed genes; **(D)** The heatmap revealed the expression level of immune-related genes in glioma cohort.

Differential expression analysis was performed between the groups with high and low TAM levels in the TCGA-GBM cohort to identify genes associated with TAM.A total of 2,311 genes were identified as differentially expressed using a log^2^ FC threshold of 0.585 and a P-value threshold of 0.05.Out of these, a total of 1,194 genes experienced down-regulation, whereas 1,117 genes underwent up-regulation.Furthermore, immune-associated genes were acquired from an online repository, leading to a grand total of 1,793 immune-associated genes (**Figures 5A, B**). By comparing these with TAM-related genes, 350 genes were found to be involved in both categories and were further analyzed. To visualize the expression levels of TAM-associated genes and immune-associated genes in the TCGA-GBM cohort, heatmaps were created (**Figures 5C, D**).

### Developing the risk model for TAM in the TCGA-GBM cohort

**Figure 6:**
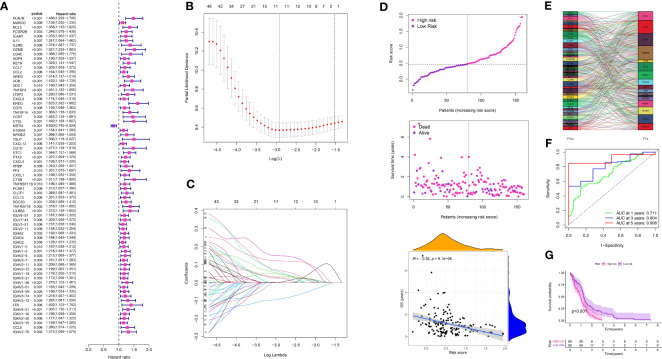
**(A)** The univariate COX regression analysis in glioma cohort; **(B, C)** The LASSO regression analysis in glioma cohort; **(D)** The risk plot in glioma cohort; **(E)** The correlation analysis between transcription factors and prognosis-related genes; **(F)** The time-dependent ROC curve in glioma cohort; **(G)** The survival analysis between low- and high-risk groups.

Using the 350 genes associated with TAM, a risk model was built in the TCGA-GBM cohort. Prognostic genes were identified through the utilization of COX regression analysis and LASSO regression analysis. Using a p-value threshold of 0.01, a univariate COX regression analysis identified 71 genes that exhibited a significant correlation with prognosis in glioma (**Figure 6A**). LASSO regression analysis further narrowed down the selection to 12 prognosis-related genes (**Figures 6B, C**). Based on these genes, a risk score was allocated to every glioma patient, and subsequently, the patients were categorized into low- and high-risk groups by utilizing the median risk score (**Figure 6D**). A risk chart was created to display the spread of risk scores in the glioma group. Furthermore, an assessment was conducted to determine the association between transcription factors and genes related to prognosis, revealing a strong connection between the two (**Figure 6E**). The risk model’s predictive value was evaluated by analyzing ROC curves, which yielded AUC scores of 0.711, 0.804, and 0.906 for survival rates at 1 year, 3 years, and 5 years, respectively (**Figure 6F**). The survival analysis indicated that individuals in the high-risk category experienced worse overall survival in comparison to those in the low-risk category (**Figure 6G**).

### The risk model associated with TAM shows a strong correlation with cells related to the immune system

**Figure 7:**
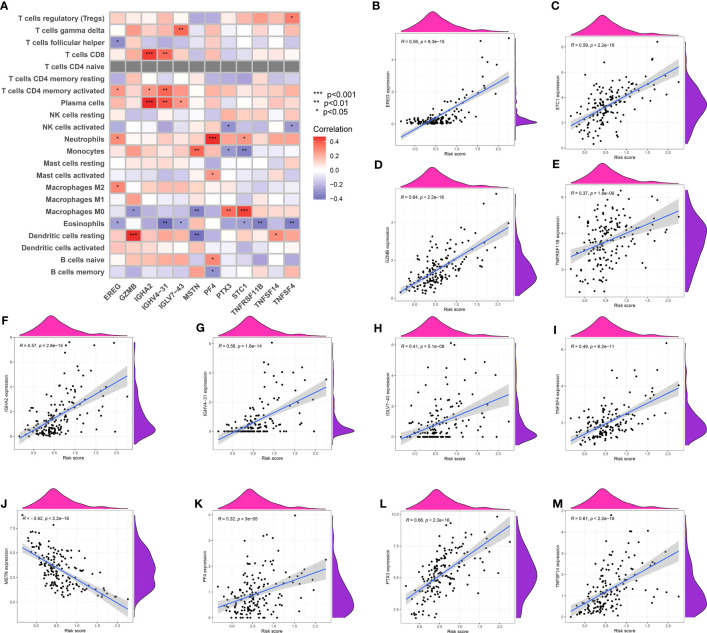
**(A)** The correlation analysis between immune-related cells and genes involved in risk model; The correlation analysis between risk score and EREG **(B)**, STC1 **(C)**, GZMB **(D)**, TNFRSF11B **(E)**, IGHA2 **(F)**, IGHV4-31 **(G)**, IGLV7-43 **(H)**, TNFSF4 **(I)**, MSTN **(J)**, PF4 **(K)**, PTX3 **(L)** and TNFSF14 **(M)**.

The risk model associated with TAM exhibited a notable correlation with cells related to the immune system. The risk model showed strong associations with particular immune-related cell types, involving a total of 12 genes. EREG was linked to M2 macrophages, eosinophils, and neutrophils, whereas GZMB exhibited a robust reaction to resting dendritic cells and M0 macrophages. IGHA2 showed a correlation with plasma cells, activated T memory cells, and CD8 T cells. MSTN exhibited connections with quiescent dendritic cells, M0 macrophages, and monocytes, whereas PF4 demonstrated enrichment in memory B cells, naive B cells, activated mast cells, and neutrophils (**Figures 7A–M**).

### Determining the association between the risk model and crucial genes

**Figure 8:**
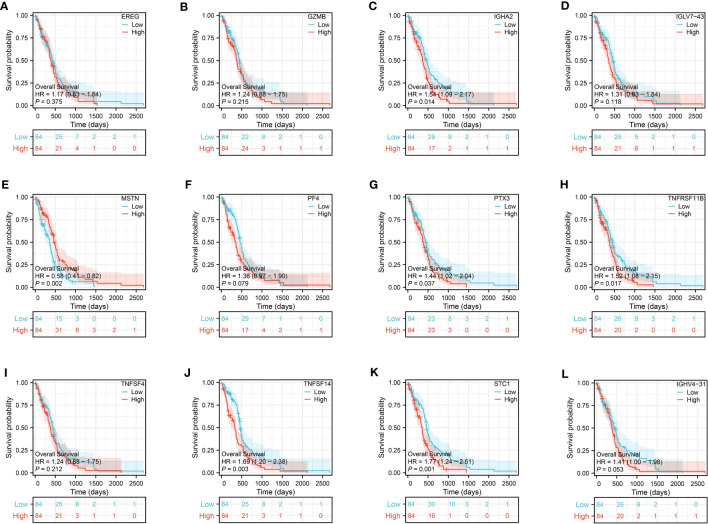
The survival analysis of EREG **(A)**, GZMB **(B)**, IGHA2 **(C)**, IGLV7-43 **(D)**, MSTN **(E)**, PF4 **(F)**, PTX3 **(G)**, TNFRSF11B **(H)**, TNFSF4 **(I)**, TNFSF14 **(J)**, STC1 **(K)** and IGHV4-31 **(L)** in glioma cohort.

The correlation analysis between the risk score and key genes in the risk model showed predominantly positive correlations, suggesting that higher risk scores were linked to elevated gene expression of EGER, STC1, GZMB, TNFRSF11B, IGHA2, IGHV4-31, IGLV7-43, TNFSF4, PF4, PTX3, and TNFSF14. However, MSTN showed a negative correlation with the risk score (**Figures 8A–L**).

### The predictive value of genes in risk model

**Figure 9:**
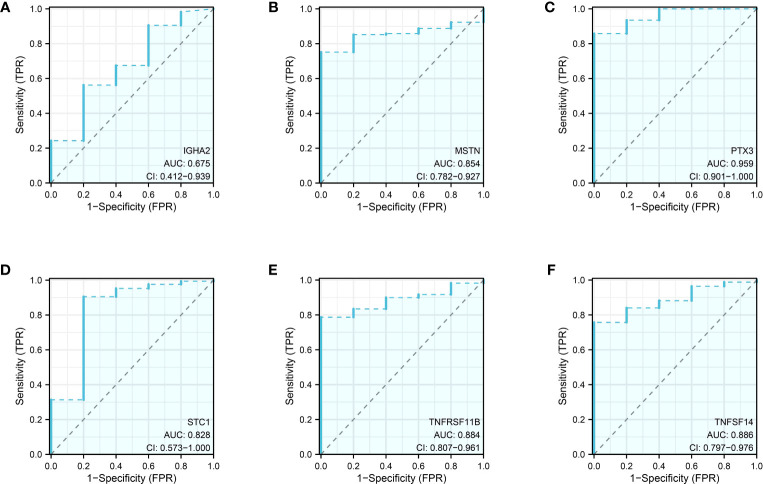
The ROC curve of IGHA2 **(A)**, MSTN **(B)**, PTX3 **(C)**, STC1 **(D)**, TNFRSF11B **(E)** and TNFSF14 **(F)** in glioma cohort.

Prognosis prediction based on the risk model revealed that IGHA2, MSTN, PTX3, TNFRSF11B, STC1, and TNFSF14 exhibited significant prognostic value with p-values below 0.05 during the survival analysis of the 12 genes. In the glioma cohort, the predictive value of these genes was further confirmed through time-dependent ROC curve analysis, yielding AUC scores of 0.675, 0.854, 0.959, 0.828, 0.884, and 0.886 (**Figures 9A–F**).

### The potential pathways of PTX3 in glioma cohort

**Figure 10:**
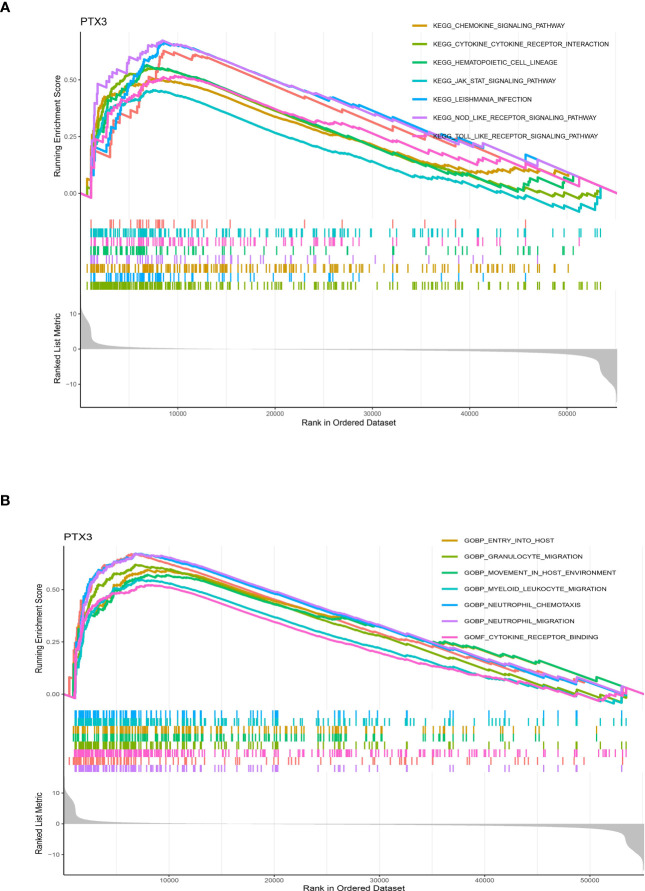
**(A)** The GSEA in KEGG terms; **(B)** The GSEA in GO terms.

Pathway analysis of PTX3, which exhibited potential as a prognostic indicator, unveiled enrichment in KEGG categories like cytokine-receptor interaction, chemokine signaling, and hematopoietic cell lineage (**Figure 10A**). Additionally, GO terms related to neutrophil migration, acute inflammatory response, cytokine receptor binding, lymphocyte-mediated immunity, cell-substrate junction, and macrophage activation were found to be significantly enriched (**Figure 10B**).

### Enhanced PTX3 expression may stimulate the growth capacity of glioma cells

**Figure 11:**
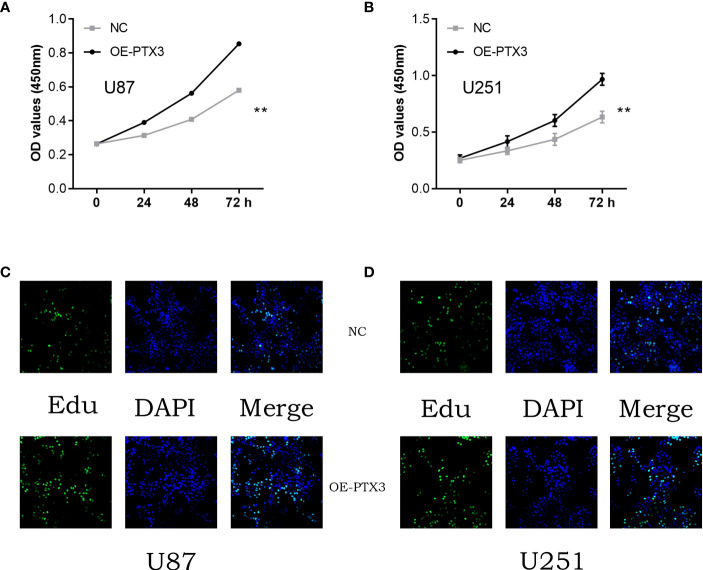
**(A)** The cck8 assay in U87 cells; **(B)** The cck8 assay in U251 cells; **(C)** The Edu assay in U87 cells; **(D)** The Edu assay in U251 cells.

In order to assess the glioma cohort’s cell proliferation and invasion capability of PTX3, we subsequently conducted experiments on glioma cells to measure their cell proliferation ability. According to the CCK8 assay and EDU assay, it was found that the excessive expression of PTX3 enhanced the glioma cells’ capacity for cell proliferation (**Figures 11A–D**).

## Discussion

Glioma, a malignant tumor originating in the central nervous system, is both the most prevalent and the most aggressive form, often leading to a grim prognosis. The most malignant form of glioma, known as grade IV, was classified by WHO (18). Gliomas still have a poor outcome following active surgery combined with radiotherapy and chemotherapy (19). Understanding the molecular mechanisms of glioma progression is crucial for enhancing patient prognoses, making it highly significant (20). Signaling abnormalities associated with gliomas extend far beyond cancer cells, affecting the interactions between TMEs as well (21). In glioma (22), the TME contains a large number of bone marrow-derived macrophages and brain microglia, making up the TAM, which is the predominant cell population (23). New treatment options could potentially arise from gaining a more comprehensive comprehension of the intricate interplay between gliomas and TAMs. Our objective in this study is to investigate the possible relationship between TAM and GBM. Initially, the glioma cohort underwent single-cell RNA analysis to assess the level of gene expression in various cells. The genes related to TAM were considered, including C11orf45, CD68, CLEC5A, CYBB, FUCA1, GPNMB, HS3ST2, LGMN, MMP9, and TM4SF19, which are markers associated with macrophages. Then, to further obtain the TAM-related genes in glioma cohort, we also performed the ssGSEA analysis. The TCGA-GBM cohort was categorized into low- and high-TAM groups based on the median score related to macrophages. Additionally, a comparative analysis was conducted between the low-TAM and high-TAM groups to identify differentially expressed genes. After identifying the genes with differential expression, we proceeded to conduct both COX regression analyses in order to obtain the genes associated with prognosis in the risk model. The glioma cohort demonstrated the significant predictive power of the TAM-related model through survival analysis and ROC curve. In recent times, numerous research investigations have concentrated on examining the significance of TAM in the glioma cohort (24). A study has found that exosomes can activate the JAK2/PI3K/Akt pathway by targeting CBLB, promote macrophage polarization to the M2 phenotype, and subsequently promote tumor progression (25). In this study, we have demonstrated the significance of the TAM-associated risk model in a cohort of glioma patients. Also, the 12-genes based risk model can be regarded as a useful tool in the treatment, prognosis and prediction in glioma cohort. Over the past few years, numerous research studies have developed a risk model that is associated with prognosis. This model aims to enhance the accuracy of predicting the diagnosis, treatment, and prognosis. SYT16, identified through analysis of the TCGA data, was determined to be a glioma prognostic immune biomarker (26). Furthermore, examination of individual-cell sequences and transcriptome profiles reveals ubiquitination-related patterns in glioma and discovers USP4 as a new biomarker (27).

Studies have demonstrated that M2 macrophages exhibit PD-L1, which is among the immune checkpoint inhibitors employed for the regulation of macrophages. Exosomes facilitate the induction of M2 macrophages and PD-L1 expression in human monocytes by GBM cancer stem cells, as reported in a prior investigation (28). Over time, TAMs in mouse malignancy models consistently exhibit an elevated expression of PD-1, which is also observed in the progression of human cancer stages (29). In this study, we conducted a correlation analysis between genes associated with immune checkpoints and groups related to TAMs. The findings indicated a strong correlation between the TAM-associated scores and the expression level of genes related to immune checkpoints. The varying levels of expression of genes related to immune checkpoints could potentially provide guidance for immune checkpoint therapy in individuals diagnosed with GBM.

Furthermore, we conducted a pathways enrichment analysis using the genes associated with TAMs in the glioma cohort. Many immune-related pathways were significantly enriched. Despite advancements in surgical techniques and clinical protocols, the management of high-grade gliomas continues to pose challenges, characterized by low rates of treatment success and short survival times (30). Gliomas present several challenges, including their molecular complexity and their inconsistency in histopathological grade. Inaccurate forecasts of disease advancement and the ineffectiveness of conventional treatments can be caused by these variables. Currently, the current standard treatment for patients with glioma involves the use of both postoperative radiotherapy and adjuvant chemotherapy (31). However, ongoing efforts are being made to develop alternative methods of treating glioma patients who are unresponsive to treatment or experience recurrence following completion of treatment.

While bioinformatics analyses provide powerful tools for generating insights from large-scale datasets, they are not without limitations (32). These computational methods inherently depend on the quality and accuracy of the input data (33). Hence, any errors, biases, or variations in the initial data can significantly influence the results and interpretations (34). This reliance on the quality of input data is particularly relevant for analyses involving next-generation sequencing, where factors such as sequencing depth, quality of reads, and sample preparation methods can introduce biases (35). Another limitation is the assumption of linearity in many bioinformatics models, which may not adequately represent the complex, non-linear biological systems in reality (36). Moreover, many bioinformatics tools rely on existing databases, the contents of which are continually evolving. Therefore, results from such tools are constrained by the current state of knowledge and can become outdated as new data are added to these databases.

By utilizing bioinformatics analysis, we have effectively developed the TAM-associated risk model in the glioma cohort. Afterwards, the additional examination uncovered the significant forecasting significance of the TAM-associated risk model in the glioma group. The analysis effectively offered a fresh approach to the care and identification of individuals with glioma.

## Data availability statement

The original contributions presented in the study are included in the article/supplementary material. Further inquiries can be directed to the corresponding author.

## Author contributions

The author contributions for YNH, WJQ, YGL, XLQ, JL, HY, and LZC are as follows: YNH: Conceptualization, Methodology, Writing - Original Draft. WJQ: Data Curation, Software, Validation. YGL: Formal Analysis, Investigation. XLQ: Resources, Data Curation. JL: Supervision, Project Administration. HY: Writing - Review & Editing, Visualization. LZC: Funding Acquisition, Supervision. All authors contributed to the article and approved the submitted version.
